# Virtual conference participant’s perceptions of its effectiveness and future projections

**DOI:** 10.1186/s12909-021-03040-9

**Published:** 2022-01-03

**Authors:** Kyong-Jee Kim, Seo Rin Kim, Jangwook Lee, Ju-Young Moon, Sang-Ho Lee, Sung Joon Shin

**Affiliations:** 1 Department of Medical Education, Dongguk University School of Medicine, Goyang, South Korea; 2 Department of Nephrology and Research Institute for Convergence of Biomedical Science and Technology, Pusan National University Yangsan Hospital, Yangsan, South Korea; 3 Department of Internal Medicine, Dongguk University Ilsan Hospital, Goyang, South Korea; 4 Division of Nephrology, Department of Internal Medicine, Kyung Hee University, College of Medicine, Seoul, South Korea; 5 Department of Internal Medicine, Dongguk University School of Medicine, Goyang, 10326 South Korea

**Keywords:** Virtual conference, Perception, COVID-19

## Abstract

**Background:**

The virtual conference format has become an essential tool for professional development of researchers around the world since the outbreak of the COVID-19 pandemic. This study aims to identify empirical evidence of the benefits and challenges of virtual conferences by investigating participants’ experiences with them.

**Methods:**

The study participants were delegates to the 40th annual meeting of the Korean Society of Nephrology, which was held virtually in September, 2020. A questionnaire was developed and implemented among the conference attendees. The 44-item questionnaire included five sub-scales related to participant perceptions of the virtual conference, which were (a) convenience and accessibility, (b) planning and organization, (c) technology use, (d) social exchanges, and (e) overall satisfaction, their preferences of conference formats, and their views of future projections for a virtual conference.

**Results:**

A total of 279 delegates completed and returned the questionnaires (18.8% response rate). Participants varied in gender, age, profession, work location, and prior experience with conferences. On a four-point Likert scale (1 = “strongly disagree” and 4 = “strongly agree”), participants showed positive perceptions of the virtual conference in general, where the total mean (M) was 3.03 and less positive perceptions on social exchanges (M = 2.72). Participant perceptions of the virtual conference differed across age groups, professions, and prior experience with conferences (*p* < .05). Approximately half of the participants (*n* = 139) preferred the virtual format, and 33% (*n* = 92) preferred the conventional format. Participant preferences for the virtual format were somewhat evenly distributed between asynchronous (32.9%) and synchronous (29.1%) modes. Participants predicted a virtual conference would continue to be a popular delivery format after the end of the COVID-19.

**Conclusions:**

Although participants had positive perceptions of the virtual conference, more support needs to be offered to those who may be less comfortable with using technology or with online interactions, and there is a need for improvement in supporting social exchange among attendees. Also, it is suggested that a blend of asynchronous and synchronous delivery methods should be considered to meet the varied needs of attendees.

## Background

We have seen abrupt transitions of scientific meetings into a virtual format worldwide since the outbreak of the COVID-19 pandemic [1, 2]. Online delivery of scientific meetings has been in place for some time, such as in webinars [3], but the travel restrictions caused by the pandemic accelerated the delivery transition. Virtual meetings have benefits over conventional face-to-face meetings, including the saving of participants’ time and reducing the cost for travel to the conference venue [4, 5], which also contributes to reducing the environmental burden associated with long-distance travel [6, 7]. Consequentially, the virtual conference format has become a critical tool for researchers’ professional development worldwide in a time when in-person conference meetings are curtailed.

Virtual conferences offer worldwide researchers several benefits. The virtual format allows conference organizers to increase the number of conference participants markedly [2]. Virtual meetings can make conferences more inclusive for those who are otherwise unable to travel to international conferences, such as those disabled or have health problems, and members of under-developed scientific communities [8]. Speakers from distant locations can participate more willingly in virtual conferences, which improves the quality and diversity of program [9]. As organizers make efforts to make conference meetings as inclusive and diverse as possible by utilizing the virtual format, those efforts can also present several challenges. Among such challenges, delivering an online synchronous session presents hurdles for participants who are spread across many time zones, and there are limited abilities to interact with speakers in real-time [10]. Moreover, it is often difficult to sustain attendees’ undivided attention in an online setting [11]. Disadvantages of virtual ones include lack of traditional professional networking and social interactions. The non-verbal communication, fine nuance, and mutual rapport from in-person communication cannot be replaced easily by online format [12]. The virtual setting might have less sponsorship options and divest some participants of tourism opportunity [9]. In addition, hands-on training and workshops are hardly hosted in virtual conferences, which leads to a compromised program variety [9].

Although virtual conferences have become very popular since the pandemic outbreak, there is little empirical evidence of their effectiveness. Given the several benefits and challenges of virtual conferences, research providing an empirical understanding of the design and implementation of effective virtual conferences is warranted. Thus, the research questions for this study were:

1. What are participant perceptions of virtual conferences?
2. What are participant predictions for virtual conferences in the future?
3. Do participant perceptions of virtual conferences vary with demographic and background factors?

By investigating conference participants’ experiences with virtual conferences, this study intends to identify empirical evidence of the benefits and challenges of virtual conferences, which have implications for improving its quality.

## Materials and methods

### Study participants and settings

Study participants were delegates to the 40th annual meeting of the Korean Society of Nephrology (KSN) held on September 25-27, 2020. Before the pandemic, the KSN annual meetings had taken place in a conventional format, with an average of 2000 international delegates attending. The conference participants are health professionals and researchers in the field of nephrology and include physicians, nurses, and scientists. The annual KSN meeting was transformed to an entirely virtual conference format in 2020, and 2177 international delegates registered for the program. All sessions in the program, which included plenary lectures, oral abstracts, and poster presentations, were delivered online. The presentations were delivered either synchronously or asynchronously, and Q&A sessions, using either audio or text messages, followed the synchronous online sessions.

### Instrument and procedures

The authors developed the questionnaire for this study by collaborating with expert researchers in related fields. The items in the questionnaire were developed following a review of relevant literature [1–10]. Three researchers, who were active members of the KSN and had attended the annual conferences for over a decade, reviewed and checked content validity. Another researcher who is involved in medical education and has relevant expertise participated in the design and implementation of the questionnaire. She also had a main role in the literature review and drafted the questionnaire. In addition, a biostatistician reviewed the questionnaire and provided feedback to improve its validity. In addition, a pilot survey was conducted using three conference attendees to check for item clarity, and based on the results, the necessary changes were made.

The questionnaire consisted of three sections comprising 44 items. The six items in the first section solicited respondent demographic information, and another six items were related to their attendance type (i.e., presenter, attendance without any presentation, location and duration of attendance, and technology used for attendance). The second section included 20 items related to respondent perceptions of the virtual conference, and the items were assessed using the 4-point Likert scale (1 = ‘strongly disagree’ and 4 = ‘strongly agree’). The items on respondent perceptions of virtual conferences encompassed five sub-scales identified during the literature review: (a) convince and accessibility, (b) planning and organization of the conference, (c) technology use, (d) social exchanges, and (e) overall satisfaction. This section also included an item related to their preferences for conference format (virtual or conventional) that was assessed using a 7-point Likert scale (1 = ‘strongly favor the conventional format’ and 7 = ‘strongly favor the virtual format’) and two items related to their preference between asynchronous and synchronous modes within the virtual conference format. In the third section, 7 items solicited respondent predictions on the format of scientific meetings in the future using a 4-point Likert scale (1 = ‘strongly disagree’ and 4 = ‘strongly agree’). The instrument also included two open-ended questions on respondent perceptions and future predictions of virtual conferences.

### Data collection and ethical considerations

Participation was solicited by sending an e-mail to conference delegates who agreed to receive an e-mail from the conference organizer. Among the 2177 delegates in the KSN meeting held in 2020, international delegates (*n* = 279) were excluded from this study as the questionnaire was in Korean; thus, the e-mail was sent to domestic delegates only. The questionnaires were active for 1 month during October and November, 2020, 1 month after the conference had ended. A reminder e-mail was distributed 2 weeks after the initial e-mail with the link to the survey website to reduce nonresponse bias by encouraging participant response [13].

Questionnaires were administered after acquiring permission from the Institutional Review Board of Dongguk University Ilsan Hospital (IRB # DUIH 2020-10-039-001). The researchers provided all participants with a description of the purpose and methods of the study, stressed their rights regarding voluntary participation in the study, and assured them of personal confidentiality.

### Data analysis

Descriptive analysis was performed on data regarding respondents’ demographics and backgrounds, their perceptions of the conference, and their projections for future virtual conferences. Data were presented as numbers and percentages for categorical variables. Continuous data were expressed as mean ± standard deviation (SD) or median with Inter Quartile Range (Q1:Q3). The Kolmogorov-Smirnov test was used to assess normality, and the Mann-Whitney U-test and Kruskal Wallis tests were employed to compare participant responses across demographic and background categories. The post-hoc Dunn test was employed following the detection of a significant Kruskal Wallis test result. The internal consistency of items was evaluated using Cronbach’s alpha. The data were analyzed using SPSS version 27 for Windows (IBM Corp., Armonk, USA), and statistical significance was accepted for *p* values < 0.05.

## Results

### Respondent demographic information and attendance format

A total of 279 delegates completed and returned the questionnaires, representing an 18.8% response rate. Table 1 shows respondent demographic and background characteristics. Participants varied in gender, ages, professions, and work locations. The participants also varied in the number of annual KSN meetings that they had attended previously. In terms of participants’ experiences with virtual conferences, approximately 70% of participants had prior experience, while 30% had no experience with them.

**Table 1:** Respondent demographics and backgrounds

Categories	Variables	Number of respondents (%)
Sex	Male	144 (51.6)
	Female	135 (48.4)
Age	Sixties and over	21 (7.5)
	Fifties	55 (19.7)
	Forties	122 (43.7)
	Thirties	77 (27.6)
	Twenties	4 (1.4)
Professions	Professors	128 (45.9)
	Employed doctors	70 (25.1)
	Independent doctors	35 (12.5)
	Fellows	20 (7.2)
	Residents	4 (1.4)
	Nurses	10 (3.6)
	Others	12 (4.3)
Work locations	Seoul metropolitan area	154 (55.2)
	Yeongnam	72 (25.8)
	Chungcheong	27 (9.7)
	Honam	21 (7.5)
	Gangwon and Jeju	6 (1.8)
Attendance in the annual meetings	Over 21 years	43 (15.4)
	11-20 years	92 (33)
	6-10 years	65 (23.3)
	1-5 years	63 (22.6)
	First time	16 (5.7)
Prior experience with virtual conference	Yes	197 (70.6)
	No	82 (29.4)
Attended conference as a presenter	Yes	136 (48.7)
	No	143 (51.3)

Note: Others include researchers, pharmacists, students, nutritionists, or business employees

Approximately half of the respondents (*n* = 143, 51.3%) attended the conference as a non-presenter, while 136 (48.7%) were presenters for plenaries, oral abstracts, and posters. In the multiple response questions, 53.8% of the responders answered that they used a personal computer (*n* = 150), which were followed by smartphones (*n* = 116, 41.6%), laptops (*n* = 93, 33.3%), and tablets (*n* = 33, 11.8%) for attendance. Approximately two-thirds (*n* = 186) of the respondents attended the conference from their workplace, 63.8% (*n* = 176) attended from home, others attended while on a ride (67), and 2 attended from other places (coffee shop and hotel). Over the 3 day duration of the conference, 112 respondents (40.1%) attended 50-79% of the all sessions, 89 (31.9%) did more than 80%, 53 (19%) of 20 - 49%, and 25 (9%) of less than 20%.

### Respondent perceptions of virtual conference

Table 2 summarizes the descriptive statistics for respondent perceptions of the virtual conference. Respondents showed positive perceptions of the convenience and accessibility of the virtual conference (Med = 3.0 (2.75:3.25)), and they were positive about the quality of planning and organization of the conference (Med = 3.0 (2.83:3.33)). Participants slightly agreed that there were sufficient opportunities for social exchanges among participants (Med = 2.67 (2.67:3.0)). Participant attitude toward the use of technology, including difficulties in using information technology, the Internet connection, and proper technical assistance, was also favorable (Med = 2.67 (2.67:3.0)), although this is a limited finding due to insufficient scale reliability (Cronbach’s α = 0.61). Participants were generally satisfied with the virtual conference (Med = 3.0 (3.0:4.0)), with excellent internal consistency of items (Cronbach’s α = 0.94).

**Table 2:** Descriptive statistics of respondent perceptions of the virtual conference and Cronbach’s α (*n* = 279)

Sub-scales	Mean (SD)	Median (Q1:Q3)	Cronbach’s α
Overall satisfaction	3.23 (.63)	3.0 (3.0:4.0)	.94
Planning and organization	3.05 (.43)	3.0 (2.83:3.33)	.81
Technology use	3.03 (.50)	2.67 (2.67:3.0)	.61
Convenience and accessibility	3.02 (.59)	3.0 (2.75:3.25)	.74
Social exchanges	2.75 (.58)	2.67 (2.67:3.0)	.73
Total	3.03 (.73)	2.9 (2.70:3.25)	

Note: Items were rated using a four-point Likert scale, where 1 = “Strongly disagree” and 4 = “Strongly agree.” One item (“There were difficulties in using IT during the conference.”) with a negative connotation was calculated after being reverse coded

Participants showed differences in their overall perceptions of the virtual conference across their backgrounds. Younger participants had more positive perceptions of the virtual conference than their older counterparts (H = 13.89, *p* < .01), and the longer they had attended the annual meetings, the less positive perceptions they had of the virtual conference (H = 30.67, *p* < .01). In more detail, the younger participants showed more positive perceptions in the convenience and accessibility, planning and organization, social exchange, and technology use scales (*p* < .05), but they did not show any difference in the overall satisfaction scale (*p* = .19). Furthermore, those who had prior experience with a virtual conference perceived it more positively (Z = 2.48, *p* < .05). Participant perceptions of the virtual conference also differed across professions; residents, fellows, and practicing doctors showed more positive overall perceptions than those of medical faculty (H = 22.63, *p* < .001). More specifically, participants differed in their perceptions across professions in the convenience and accessibility, planning and organization, social exchange, and overall satisfaction scales (*p* < .05), but did not differ in the technology use scale (*p* = .09). Still, there were no differences in participants’ overall perceptions across genders (Z = 1.88. *p* = .06).

**Fig. 1:**
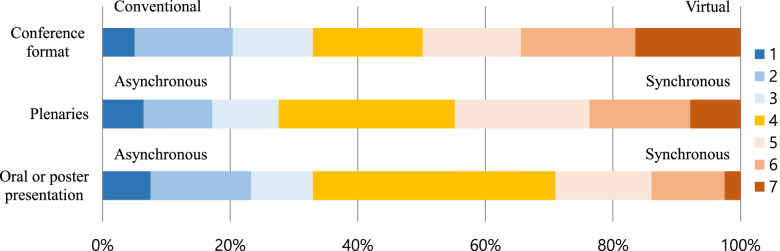
Respondent preferences for conference format (*n* = 279). Items were rated using a seven-point Likert scale, where 1 = “Strongly prefer conventional / asynchronous format” and 7 = “Strongly prefer virtual / synchronous format”

Figure 1 represents participant preferences of the conference format. Approximately half of the participants (*n* = 139) preferred the virtual format, while 33% (*n* = 92) preferred the conventional format. Participant preferences for the virtual conference format were somewhat evenly distributed between asynchronous (*n* = 92, 32.9%) and synchronous (*n* = 81, 29.1%) modes, while 38% (*n* = 106) did not express any mode preference. Moreover, participant preferences for the virtual conference format differed slightly according to presentation type. For the delivery of plenaries, participants preferred the synchronous format (125, 44.8%) over the asynchronous mode (*n* = 77, 27.6%). For oral or poster presentations, 33% (*n* = 92) of the participants preferred the asynchronous format, and 29% (*n* = 81) favored the synchronous mode.

Participant preferences of the conference format differed across professions, where residents, fellows, and practicing doctors preferred the virtual format over the conventional one more than medical faculty participants did (H = 48.60, *p* < .001). Still, participants did not differ across professions in their preferences of the format of the virtual conference (i.e., asynchronous or synchronous modes) regardless of presentation type (H = 3.98, *p* = .70 for plenaries, and H = 9.65, *p* = .14 for oral abstracts or posters). Participant preferences of the conference format did not differ across age groups (H = .19, *p* = 1.00) or genders (Z = 1.31, *p* = .95). Nor did they differ in their preferences of the conference format between the group who had prior experience with a virtual conference and the group without such an experience (Z = .93, *p* = .36).

### Respondents’ future projections of virtual conferences

Table 3 shows respondent projections for the future of virtual conferences. Respondents predicted that virtual conferences would become more popular (Med = 3.0 (3.0:4.0)). More than half of them (*n* = 165, 59.2%) disagreed with the statement that the need for a virtual conference would decrease after COVID-19 (Med = 2.0 (2.0:3.0)). They agreed that the virtual conference format would lead to more convenience and accessibility and help the meetings become more active and cost-effective (all Med = 3.0 (3.0:4.0)). A striking majority of the respondents (*n* = 272, 97.5%) agreed with the importance of technology for the successful implementation of virtual conferences (Med = 4.0 (3.0:4.0)). Meanwhile, over half of respondents (*n* = 148, 53%) agreed that there are still technical barriers to the successful delivery of a virtual conference (Med = 3.0 (2.0:3.0)).

**Table 3:** Respondent projections of the future of virtual conferences

Items	Mean (SD)	Median (Q1:Q3)
The use of technology will become more important for the successful delivery of conferences.	3.47 (.56)	4.0 (3.0:4.0)
Virtual conferences will become more popular.	3.26 (.60)	3.0 (3.0:4.0)
Researchers will be able to attend the conference with more ease and convenience in the virtual format.	3.23 (.64)	3.0 (3.0:4.0)
Virtual conferences will be more cost-effective than conventional ones.	3.18 (.65)	3.0 (3.0:4.0)
The virtual conference will help revitalize the academic society.	3.11 (.74)	3.0 (3.0:4.0)
There are still technical barriers to effective implementation of virtual conferences.	2.51 (.75)	3.0 (2.0:3.0)
The need for virtual conference will decrease when the COVID-19 ends.	2.36 (.77)	2.0 (2.0:3.0)

Note: Four-point Likert scale (1 = “Strongly Disagree” and 4 = “Strongly Agree)

## Discussion

Our study reveals the participants’ positive perceptions of the virtual conference and indicates that they predict it will continue to be a popular delivery format for conferences even after the end of COVID-19 pandemic. Still, we observed some differences in participant perceptions and preferences across the participants’ backgrounds. First, participants from outside of academia perceived the virtual conference more positively and were more likely to prefer the virtual format over the conventional one than those in academia. This finding is consistent with reports that point out the benefit of virtual conferences for widening access for researchers [8]. Second, those who were older and had more experience with conventional meetings are likely less positive about virtual conferences. This finding has a practical implication; that is, more support needs to be offered for those who are likely less comfortable with using technology or online interactions.

Our study indicates a need for improvement in supporting social exchange among attendees in the virtual conference. This finding is in line with a report indicating the limited opportunities for interaction during the virtual conference [10] and that more effective design and delivery of virtual conferences are needed to support social interactions among participants in the virtual setting. In this regard, a ‘blended conference’ format can be a solution, where a mix of face-to-face and online formats to support both those attending on-site and those participating at a distance [14], to meet the needs for social interactions among attendees. Alternative formats for a blended conference have been suggested, such as one hub and node, multi-hub and node, and multilateral hub and node [15, 16]. Additionally, the technological development facilitates computer role-playing game-type virtual physical environments with video-conferencing [17]. The recent emergence of metaverse technology might allow us to have a new ‘social’ virtual academic meeting in the foreseeable future.

Although the participants generally preferred the virtual conference over the conventional one, our study showed their preferences for the virtual conference format were heterogeneous between asynchronous and synchronous modes. This finding indicates that participants felt both asynchronous and synchronous modes had advantages and disadvantages. Therefore, it is suggested that a blend of asynchronous and synchronous delivery should be considered for an effective design and delivery of virtual conference sessions to meet the various needs of the attendees.

Participants perceived that the technical barriers interfere with the effective implementation of virtual conferences. In this study, technical issues raised included the delayed technical assistance and lagging internet connection. They joined the virtual conference using various devices, and over one third of them participated using more than two devices. Thus, it was difficult to find one presentation setup suitable for all devices. Furthermore, some participants complained of the unfamiliar conferencing platform. Addressing technical problems early, offering sufficient rehearsals, quick and proper troubleshooting, and stable internet connection and speed is warranted to overcome the barriers.

There are some limitations of the study and recommendations for future related research. First, this study was conducted among participants of a single conference. A multi-site study is warranted to enhance the generalizability of the findings from this study. Second, the low response rate of this study needs to be taken into account as it can raise the issue of non-respondent bias [13]. Although we made efforts to increase the response rate by sending the study participants several e-mails, the response rate of our questionnaires was somewhat low. Still, it is likely that our online survey method affected the response rate, which tends to be lower in online surveys than in paper-based surveys [18]. Furthermore, we compared respondent demographics with those of conference attendees, an approach used to assessing nonresponse bias [13] among age, gender, and profession groups, and detected a similarity between the two groups. Third, we used the Likert scale to measure participant opinions in this study. Although the Likert scale is an easy and popular method, it is unidimensional and has only few options as well as non-equidistant space between each choice, which might fail to measure the true attitudes of participants [19]. The Delphi technique, a process which arrives at a group opinion by surveying a panel of experts, can supplement the Likert scale and may be the option for future studies [20]. Lastly, although our study reveals participant perceptions of the current status and future of virtual conferences, it does not provide direct evidence indicating the reasons for such perceptions. Future study, using a qualitative research method, is warranted to gain a deeper understanding of participant experiences with virtual conferences.

## Conclusions

Participants had positive perceptions of the virtual conference and predicted that it would continue to be a popular format even after the end of COVID-19. Nevertheless, there is a need for improvement in supporting social exchange among attendees, technology use, and online interactions. Also, it is suggested that a blend of asynchronous and synchronous delivery methods should be considered to meet the varied needs of attendees.

## Data Availability

The datasets used and/or analyzed during the current study is available from the corresponding author on reasonable request.

## Abbreviations

COVID-19: Coronavirus disease-19
KSN: Korean Society of Nephrology
M: Mean
Med: Median
